# An extended research of crossmodal correspondence between color and sound in psychology and cognitive ergonomics

**DOI:** 10.7717/peerj.4443

**Published:** 2018-03-01

**Authors:** Xiuwen Sun, Xiaoling Li, Lingyu Ji, Feng Han, Huifen Wang, Yang Liu, Yao Chen, Zhiyuan Lou, Zhuoyun Li

**Affiliations:** 1 School of Mechanical Engineering, Xi’an Jiaotong University, Xi’an, Shaanxi, China; 2 Xi’an Gaoxin No.1 Hign School International Course Center, Xi’an, China

**Keywords:** Crossmodal correspondences, Phylosophy, Sound, Color, Speed-discrimination, Attributes

## Abstract

Based on the existing research on sound symbolism and crossmodal correspondence, this study proposed an extended research on cross-modal correspondence between various sound attributes and color properties in a group of non-synesthetes. In Experiment 1, we assessed the associations between each property of sounds and colors. Twenty sounds with five auditory properties (pitch, roughness, sharpness, tempo and discontinuity), each varied in four levels, were used as the sound stimuli. Forty-nine colors with different hues, saturation and brightness were used to match to those sounds. Result revealed that besides pitch and tempo, roughness and sharpness also played roles in sound-color correspondence. Reaction times of sound-hue were a little longer than the reaction times of sound-lightness. In Experiment 2, a speeded target discrimination task was used to assess whether the associations between sound attributes and color properties could invoke natural cross-modal correspondence and improve participants’ cognitive efficiency in cognitive tasks. Several typical sound-color pairings were selected according to the results of Experiment 1. Participants were divided into two groups (congruent and incongruent). In each trial participants had to judge whether the presented color could appropriately be associated with the sound stimuli. Result revealed that participants responded more quickly and accurately in the congruent group than in the incongruent group. It was also found that there was no significant difference in reaction times and error rates between sound-hue and sound-lightness. The results of Experiment 1 and 2 indicate the existence of a robust crossmodal correspondence between multiple attributes of sound and color, which also has strong influence on cognitive tasks. The inconsistency of the reaction times between sound-hue and sound-lightness in Experiment 1 and 2 is probably owing to the difference in experimental protocol, which indicates that the complexity of experiment design may be an important factor in crossmodal correspondence phenomena.

## Introduction

Research on crossmodal correspondences between sound and color has a long history in the field of experimental psychology. Crossmodal correspondences between color and auditory stimuli are well-established in the literature, especially in synesthetes (Karwoski, Odbert & Osgood, 1942; Cytowic, 2002; Day, 2005; Ward, Huckstep & Tsakanikos, 2006; Hänggi et al., 2008; Spector & Maurer, 2009; Menouti et al., 2015; Farina, Mitchell & Roche, 2016). For example, most synesthetes tend to associate high pitch sounds with light colors—middle ‘C’ on a piano might be red but the note three octaves higher might be green. Some studies have also compared performance to those of non-synesthetes (Ward, Huckstep & Tsakanikos, 2006). Moreover, crossmodal audiovisual mechanisms exist in the normal population more generally. There are some reviews of the literature on crossmodal correspondences in non-synesthetes (e.g., Calvert, 2001; Martino & Marks, 2001; Spence, 2011; Parise & Spence, 2013).

Early crossmodal matching studies, as well as those studies involving the matching of sound with color, suggest that people make reliable associations between certain dimensions of color and sounds. Such attributes, loudness, visual size, and hues/brightness of colors have mostly been studied in audio-visual correspondences. Marks devoted himself to studying on the correspondence between certain features of vision and hearing (Marks, 1974; Marks, 1987). He confirmed that higher pitch and louder sound were associated with lighter color. Caivano (Caivano, 1994) reported his studies about the relationship between luminosity of color and loudness of sound, saturation and timbre and size and duration based on psychological and physical parameters. Hagtvedt (Hagtvedt & Brasel, 2016) conducted three eye tracking studies on correspondence between frequency of music and lightness of colored object. In his research, participants’ visual attentions were more likely to be guided toward light-colored objects with the influence of high-frequency sounds. Some researchers also conducted similar studies on specific populations. For example, Simpson (Simpson, Quinn & Ausubel, 1956) confirmed the existence of correspondence between hue and pitch in children and concluded that high-pitch were more likely to be associated with yellow, midlevel pitch responded to orange and low-pitch matched blue. In follow-up studies, Stevens (Stevens & Marks, 1965) and Bonds (Bond & Stevens, 1969) studied both children and adults using sample waveform sounds and demonstrated that both groups with different ages matched light grey color with louder sound and darker grey color with quieter sounds. Kim (Kim, Nam & Kim, 2015) performed experiments using vowel sounds and several colors in both synesthetes and non-synesthetes. The results revealed that both synesthetes and non-synesthetes showed statistically significant color-matching consistency (e.g., high vowel sounds were mapped with brighter colors).

Also, there are some studies involving sound-color correspondence that used more complex stimuli. For example, Bresin (Bresin, 2005) selected different music to study the emotional relationship between different color attributes (hue, saturation, brightness) and music. He concluded that different hues or brightness of colors were associated to different emotional music, for instance, dark color to music in minor tonality and light colors to music in major tonality. However, we notice that the music he used was made by different musicians with various instruments, which made it impossible to identify the most influential factors among the sound properties in those music. Barbiere (Barbiere, Vidal & Zellner, 2007) did similar research and pointed out that “red”, “yellow” corresponded to happier music, whereas “gray” to sadder music. Same as Bresin’s research, four musical stimuli were studied and there were no actual colors but only words presented in the experiment. Palmer (Palmer et al., 2013) used colors instead of color-words and demonstrated that robust crossmodal matches between music and colors are mediated by emotional associations. Lindborg (Lindborg & Friberg, 2015) studied on music-color correspondence using several film music excerpts and demonstrated that happy music was associated with yellow, music expressing anger with large red color patches, and sad music with smaller patches towards dark blue.

In addition, there are some evidence which indicated that sound was also matched to other visual attributes besides colors, such as timbre and visual shapes (Adeli, Rouat & Molotchnikoff, 2014a; Adeli, Rouat & Molotchnikoff, 2014b), music and space (Salgado-Montejo et al., 2016; Hidaka et al., 2013), sound and size (Evans & Treisman, 2010; Rojczyk, 2011), or matched to other senses like gustatory attributes, such as sound and taste (Knoeferle et al., 2015; Knöferle & Spence, 2012).

Having demonstrated the ubiquitous nature of such crossmodal correspondences, the next question to be addressed by researchers is whether or not these correspondences would impact the efficacy of human information processing. Several researchers started to investigate the impact of crossmodal correspondences on human information processing using the speeded discrimination task. For instance, as one of the pioneers in this field, Bernstein in his research (Bernstein, Eason & Schurman, 1971) found that participants responded more slowly to visual stimuli when the pitch of sound in the task is inconsistent with them. Marks (Marks, 1987) did a series of discrimination experiments between vision and hearing. He pointed out that there was a strong correspondence between certain properties of vison and hearing (e.g., pitch-brightness and loudness-lightness) and concluded that subjects responded more quickly and accurately with “matching” stimuli than “mismatching” stimuli from the two modalities. In another research, Marks (Marks, Benartzi & Lakatos, 2003) also demonstrated that it was more difficult to discriminate the size of visual stimuli with the incongruent pitch of sound than with the congruent pitch of sound. In our experiments, we partly replicate the research method which Mark adopted. Other researchers also performed similar experiments in this field using the speed discrimination task (Hubbard, 1996; Marks, 1989; Melara, 1989; Gallace & Spence, 2006). Meanwhile, several researchers began to research on the underlying mechanism of this phenomenon. Hagtvedt (Hagtvedt & Brasel, 2016) studied with eye-tracking equipment and revealed that the influence was automatic without goals or conscious awareness. Evans (Evans & Treisman, 2010) demonstrated that there were strong crossmodal correspondences between auditory pitch and visual location, size, spatial frequency. He also pointed out the existence of spontaneous mapping and interaction at the perceptual level with several speeded discrimination experiments.

According to previous research (Spence, 2011), as Spence introduced in his review, the correspondences have been documented about simple stimulus dimensions, such as loudness and brightness, pitch and color-words, or more complex stimuli, such as pictures and music. There are a few restrictions when using simple waveform sounds, as we can only investigate one sound attribute in one trial. There are also some interference factors when using different music pieces, such as differences in genres, arrangements and instruments, which make it difficult to identify which sound attributes play a decisive role in correspondence. In addition, most of the previous studies have been focusing on a single property of sound, such as pitch, loudness and timbre, without comparison between different attributes. Considering the multidimensional aspects of sound, physical properties of sound and psychoacoustic (psychoacoustics is the scientific study of sound perception and audiology) of hearing determines the sound impression: pitch, tempo, sharpness, roughness, discontinuity. Pitch is the perceived frequency of sound; tempo is the speed or pace of a musical piece; Roughness belongs to psychoacoustics and it refers to the level of dissonance; sharpness is related to how much a sound’s spectrum is in the high end; discontinuity means that music lacks coherence or cohesion (Knöferle & Spence, 2012). Pitch and tempo levels are physical quantities, while roughness, sharpness and discontinuity are psychoacoustic quantities. Whether other psychoacoustic properties of sound besides pitch and tempo, such as roughness, sharpness and discontinuity, can also have associations with colors remains unknown. For previous studies on speed discrimination task, direct comparison between sound-hue and sound-lightness or other factors has never been conducted.

Both of our studies reported here are based on previous studies. In the first experiment, we investigate whether the psychoacoustics attributes (e.g., roughness, sharpness) of sound could also have significant associations with colors. Furthermore, we record and compare the reaction times when participants match each properties of sound to color. Based on the result of the first experiment, a speed discrimination task with bidirectional trails is used in the second experiment to verify whether participants’ cognitive-responses to target stimuli (color or sound) are influenced by the existence of sound-color correspondence. Participants are instructed to respond as rapidly and accurately as possible to a series of unimodal target stimuli. We compare the reaction times and error rates between congruent condition and incongruent condition, sound-brightness and sound-hue. It should be noted that this is the first study to have made a direct comparison between sound-hue and sound-lightness using the speed discrimination task.

## Experiment 1

The aim of this experiment is to replicate and extend previous findings about crossmodal correspondence between sound and color and to highlight the existence of a strong crossmodal correspondence among various attributes of sound and color, including five sound properties (pitch, roughness, tempo, sharpness and discontinuity) and three colour attributes (hue, saturation and brightness). Furthermore, we record and compare the reaction times when participants match each property of sound to color.

### Materials and methods

#### Participants

Fifty-two participants (*M*_age_ = 28.1, SD_age_ = 8.943, range 20–55 years), including 26 females and 26 males, are recruited to take part in the experiment. The number of participants are determined by G power analysis. None of them report any synesthetes experience. Given that cross-cultural differences may influence the results (Knoeferle et al., 2015), only those participants who are born in China are included in this experiment. All participants have normal color vision and sound hearing. The University of Xi’an Jiaotong University School of Medicine granted ethical approval to carry out the study within its facilities (Ethical Application Ref: No. 2017-726). All participants give their written informed consent before the start of the experiment.

#### Sound stimuli

Twenty (5 × 4) pieces of music^1^ 1All musical stimuli can be download at https://soundcloud.com/user-791045757/sets/20sounds.are created by Soundtrap online^2^ 2Soundtrap Online: https://www.soundtrap.com/., which systematically varies the five low-level properties (pitch, sharpness, roughness, discontinuity, tempo) of a 20-second piece of piano chord. The pitch is manipulated by changing the musical intervals from C2 (65.406 Hz) through C6 (1,046.5 HZ). Tempo is varied from 65 through 200 BPM. It is influenced through the application of a tremolo effect with a constant modulation frequency of 70 Hz at varying modulation amplitudes (0–100%). Sharpness is changed by applying a frequency filter that attenuated or boosted frequencies below/above 1,000 Hz from +12∕ − 12 dB through −12∕ + 12 dB. Discontinuity is manipulated by changing the decay duration of all notes from 870 through 100 ms. We use the slider in Soundtrap to increase or decrease (in several steps) the sounds’ pitch, roughness, sharpness, discontinuity and tempo. The values of each auditory parameter are set at four levels: (a) pitch: C2 (65.406 Hz), C3 (130.81 Hz), C4 (261.63 Hz) and C5 (523.25 Hz) (C5 is a high tone but is still comfortable for human ears); (b) roughness: 0%, 30%, 70% and 100%; (c) tempo: 65, 120, 150 and 200 BPM; (d) sharpness: we use 1–4 to represent the four levels of sharpness, 1 is the weakest and 4 is the strongest; (e) discontinuity: 0%, 40%, 70% and 100%. Loudness is anchored at 65 dB—a relatively comfortable sound level for human ears. Before we create the music pieces, we created a neutral tune with the value of each sound attribute set at the second lowest level (pitch: C3 (130.81 Hz); roughness: 30%; tempo: 120 BPM; sharpness: level 2; discontinuity: 40%) by Soundtrap. For each music piece, we only adjust the value of one of the five attributes, with the other four being kept at the second lowest level.

#### Color selection

**Figure 1:**
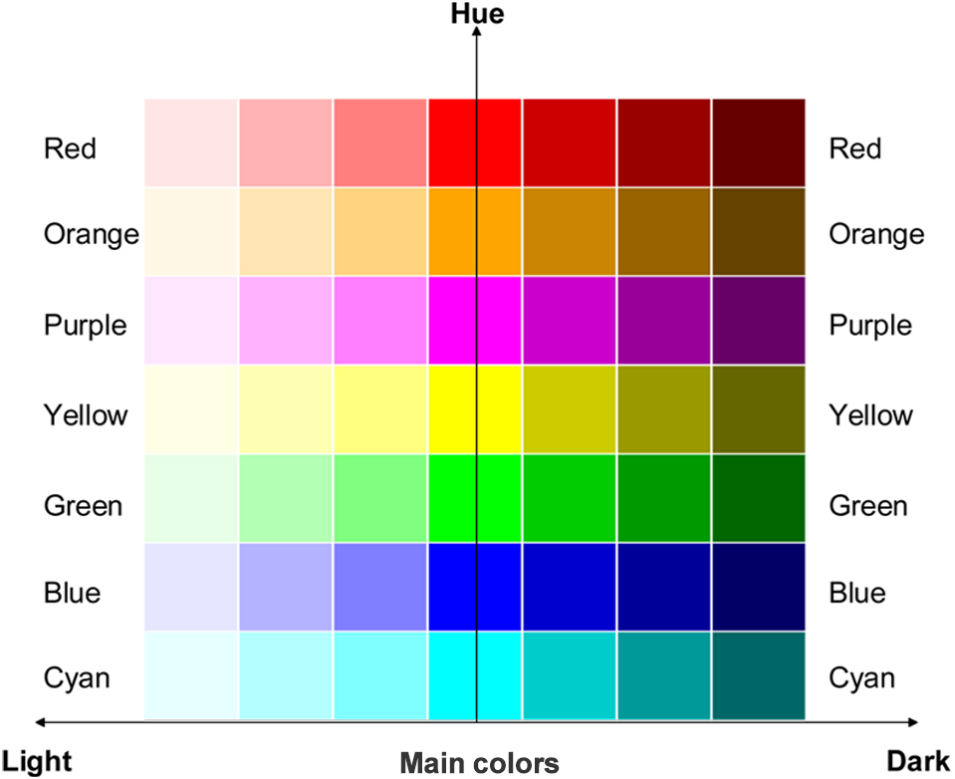
Colors used in Experiment 1. The 49 colors that were presented during the sound-color association task, including: (1) seven main colors with different hues: red, orange, purple, yellow, green, blue, cyan; (2) forty-two colors manipulated from the main colors, varied in six different levels of saturation-brightness. The vertical *y*-axis represented different hues, and the horizontal *x*-axis represented changes from light (left) to dark (right) in saturation-brightness.

Forty-nine color squares (100 × 100 pixels) are used to match the sound stimuli. Colors are coded using the Hue Saturation Brightness (HSB) scheme. Seven standard web colors with different hues, including red, orange, purple, yellow, green, blue and cyan (hex codes were FF0000, FFA500, FF00FF, FFFF00, 00FF00, 0000FF, 00FFFF, respectively), are chosen as the main colors. The other forty-two colors are manipulated by varying either the saturation or brightness values of the main colors. Saturation value is set at 40%, 60% and 80%, making the main colors lighter. Brightness value is set at 50%, 30% and 10%, making the main colors darker. Hence, all the 49 colors can also be ordered by lightness. All 49 colors are listed in Fig. 1. The background color of the experiment interface is gray (hex code AAAAAA).

#### Procedure

We develop a customized software program based on C# to administer the task in Experiment 1. Given that the experiment is performed online, the apparatus (i.e., participant’s computer and monitor) varies by participants, and participants are free to choose the place they feel comfortable to perform the experiment, either at home or at the lab, as long as it can meet our requirements of the experimental environment. We send web links to the experiment page directly to participants. Each participant is asked to prepare a headphone and be seated in front of a computer in a quiet room. We guide participants through the remote assistance to help the participants get familiar with the operation process. Before starting the main study, participants were given voice instructions and got familiar with the experimental protocol in the practice mode. In the meantime, participants are required to listen to each music piece (sound stimuli) at least twice to familiarize themselves with these music pieces. They are required to ensure the sound playback is active and to set a comfortable sound level.

**Figure 2:**
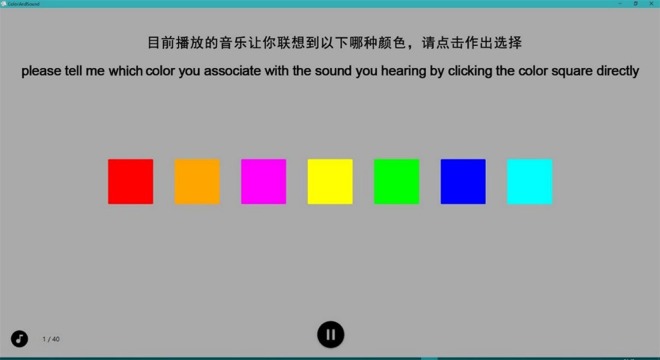
Screenshot illustrating the task used in Experiment 1. Participants should judge which of the given colors on the screen can best match the sound they hear by clicking the corresponding color square directly when they decided to choose. Participants responded with the mouse.

**Figure 3:**
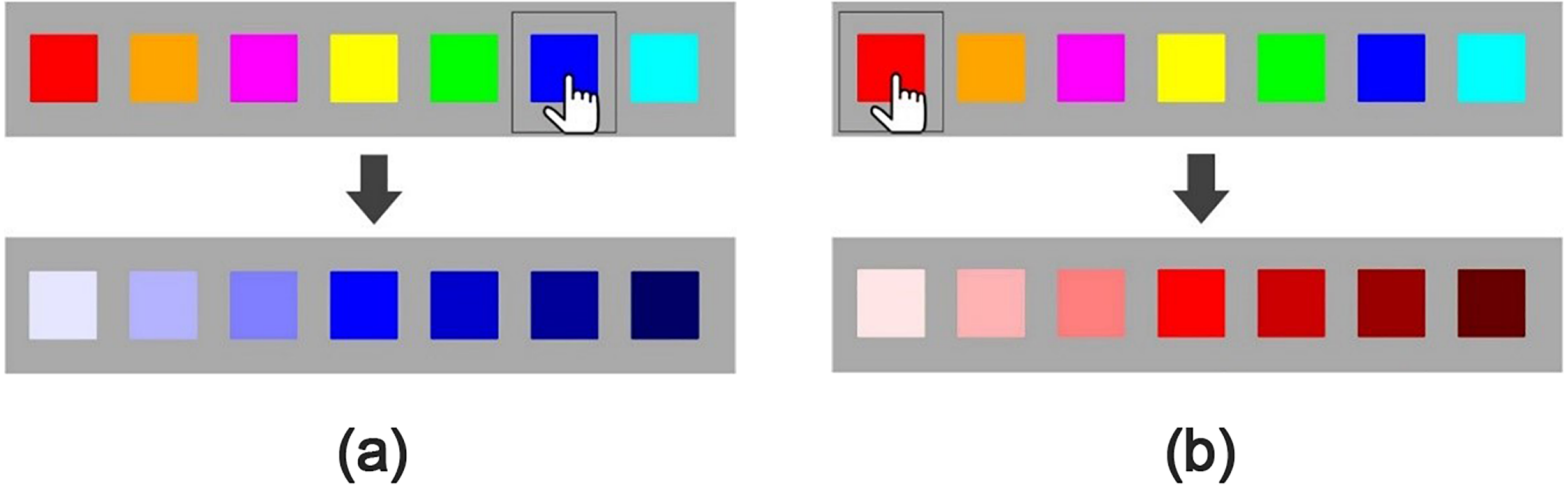
Specific explanation of the color-selection task in Experiment 1. Each trial consisted of two steps. First, participants had to judge which hue of color best matched the presented sound stimuli. Second, participants should judge which saturation-brightness of color best matched the same sound. For example, as shown in plot (A), participants choose “blue” from seven different hues to match the presented sound stimuli in the first step. Then, they should judge the saturation-brightness based on “blue” to match the same sound. Similarly, if they choose “red” in the first step, then they should judge the saturation-brightness based on red, as shown in plot (B).

After familiarization, participants click on the “start” button and the main study commence. The experiment interface is shown in Fig. 2. There are 20 trials for each participant. In each trial, participants are required to match a color to a sound stimulus. The sequence of the sound stimulus is randomized across participants, while the colors always shown in the same order. Each music piece lasts 9s. Each trial consists of three steps: the first step is a familiarization period. A white fixation point is presented at the center of the screen for an interval of 18s. In this period, we present a random sound-stimuli to participants twice so that they are able to get familiar with the sound-stimuli. Second, after the removal of the fixation point, timer is initiated. The sound stimulus continues throughout this period. Participants are shown the seven main colors (with different hues) and are asked to select the color that they feel best matched to the sound stimulus. Each music piece repeats for at most three times for participants to make choice. Third, participants are shown another group of seven color squares. The middle square is exact the same color they have selected in the first step. The other six colors have the same hue value with the middle one, but with different saturation or brightness value. For instance, participants choose blue color in the first step. Then, they are shown seven blue color squares, visually from light blue to dark blue. The brightness value the first three squares is changed to 50%, 30% and 10% respectively. The middle square is exact the same blue square as in the first step. The saturation value of the last three squares is changed to 80%, 60% and 40% respectively, as is shown in Fig. 3. Participants listen to the same sound as in the first step and made a second-round decision. Participants are required to confirm their selection by clicking on a certain color square in a limited time. Otherwise, the data would be invalid if the selection is not made within valid time. Once participants confirm and make a selection, the sound will stop and ready to start the next trial or step in 3 s. Each trial takes about 30 s∼45 s. It takes about 10∼15 min to finish the protocol. The experiment system records participants’ basic information (including name, gender, age), colors they select for best matching those sounds stimuli, the reaction time (RT) for each selection in the first step (H-RT) and the RT for each selection in the second step (L-RT) in each trial. H-RT and L-RT are recorded from the onset of the color stimuli in the second and third step, respectively, until participants make their decisions.

### Results

#### Sound-hue mappings

**Figure 4:**
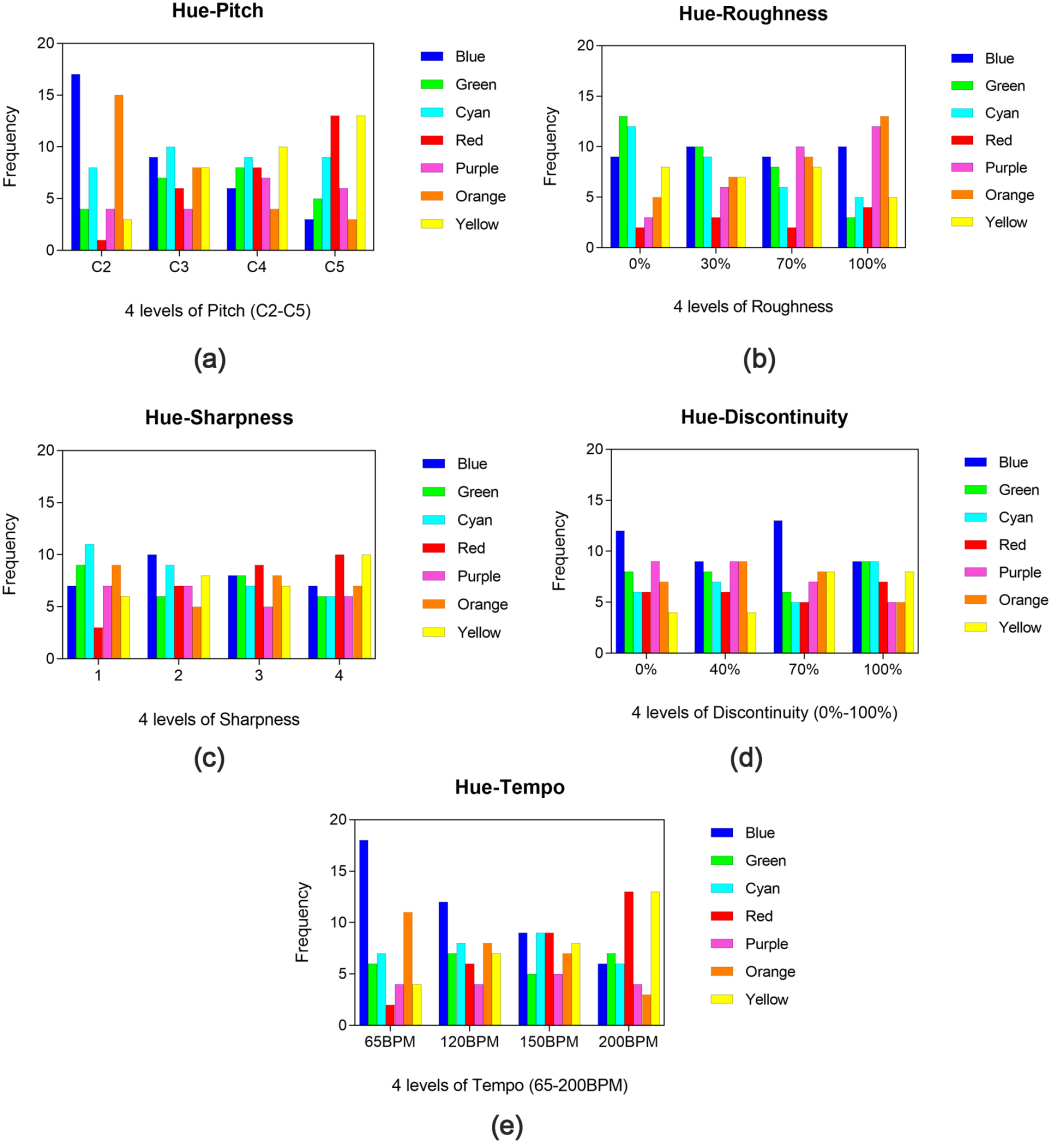
Results for sound-hue mappings. Five plots (A–E) depicting the frequency with which the different hues were selected for each of the five properties of sound (pitch, roughness, sharpness, tempo, discontinuity, in sequence). For each plot, the seven colors are shown along the *x*-axes, each color has four different values corresponding to the four levels of the property of sound. For example, in the “pitch” plot (A) and “tempo” plot (E), blue and red have four different values corresponding to the four levels of pitch (C2, C3, C4, C5) and tempo (65BPM, 120BPM, 150BPM, 200BPM). In the “roughness” plot (B), orange and green also have four different values corresponding to the four levels of roughness. But as shown in the plot (C) and (D), color selections are found to be independent from different levels of sharpness and discontinuity.

The results for sound-hue mappings are shown in Fig. 4. In order to determine whether color selections are independent from different levels of each sound attribute, chi-square test of independence is performed in SPSS. Post-hoc pairwise comparison with Bonferroni adjustment of alpha level is used to compare the difference between every two levels. Chi-square goodness of fit test is also conducted in further analysis to find out which levels of each sound attribute induce a distribution of color selection that is different from that expected by chance.

The results for pitch are shown in Fig. 4A. Results of chi-square test of independence show that color selections are significantly associated with the levels of pitch [*χ*^2^(18) = 24.192, *p* = 0.001]. Post-hoc analysis (alpha level adjusted at *α* < 0.05∕6 = 0.0083) reveals significant differences between C2 and C4 [*χ*^2^(6) = 23.053, *p* = 0.001] and between C2 and C5 [*χ*^2^(6) = 34.906, *p* < 0.001] (results for all possible combinations of pairwise comparison are listed in Table S1). Results for chi-square goodness of fit test indicate that red and yellow are most strongly linked with high pitch (C5) [*χ*^2^(6) = 15.038, *p* = 0.020], whereas blue and orange are most strongly linked with low pitch (C2) [*χ*^2^(6) = 31.462, *p* < 0.001]. When the pitch was set at C2 (lowest level), only 1.9% of the participants choose red color, while 32.7% choose blue. However, when the pitch was changed to C5 (highest level), the proportion of red color increased to 25.0%, with blue decreased to 5.7%.

The results for roughness are shown in Fig. 4B. Although color selections are found to be independent from different levels of roughness [*χ*^2^(18) = 22.356, *p* = 0.217], significant difference is found between 0 and 100% [*χ*^2^(6) = 19.500, *p* = 0.003] in post-hoc test anyway. Chi-square goodness of fit test reveals that purple and orange are associate with higher roughness [*χ*^2^(6) = 13.692, *p* = 0.033], whereas green and cyan are linked to lower roughness [*χ*^2^(6) = 14.796, *p* = 0.022].

The results for sharpness are shown in Fig. 4C. Chi-square test of independence reveals that color selections are independent from different levels of sharpness [*χ*^2^(18) = 9.508, *p* = 0.947].

The results for discontinuity are shown in Fig. 4D. Color selections are found to be independent from different levels of discontinuity [*χ*^2^(18) = 8.769, *p* = 0.965].

The results for tempo are shown in Fig. 4E. No significant difference is found in chi-square test of independence [*χ*^2^(18) = 26.717, *p* = 0.084]. However, post-hoc analysis reveals significant difference between 65 and 180 BPM [*χ*^2^(6) = 23.557, *p* = 0.001]. Results for chi-square goodness of fit test indicate that red and yellow colors are most strongly linked with fast tempo [*χ*^2^(6) = 13.154, *p* = 0.041], whereas blue and orange colors seem most strongly linked with slow tempo [*χ*^2^(6) = 24.192, *p* < 0.001]. The percentage of red color was only 3.8% while blue color was 34.6% with the tempo set at 65 BPM(slowest). When the tempo was set at 200 BPM (fastest), 23% of the participants chose red and only 9.6% chose blue.

#### Sound-lightness mappings

**Figure 5:**
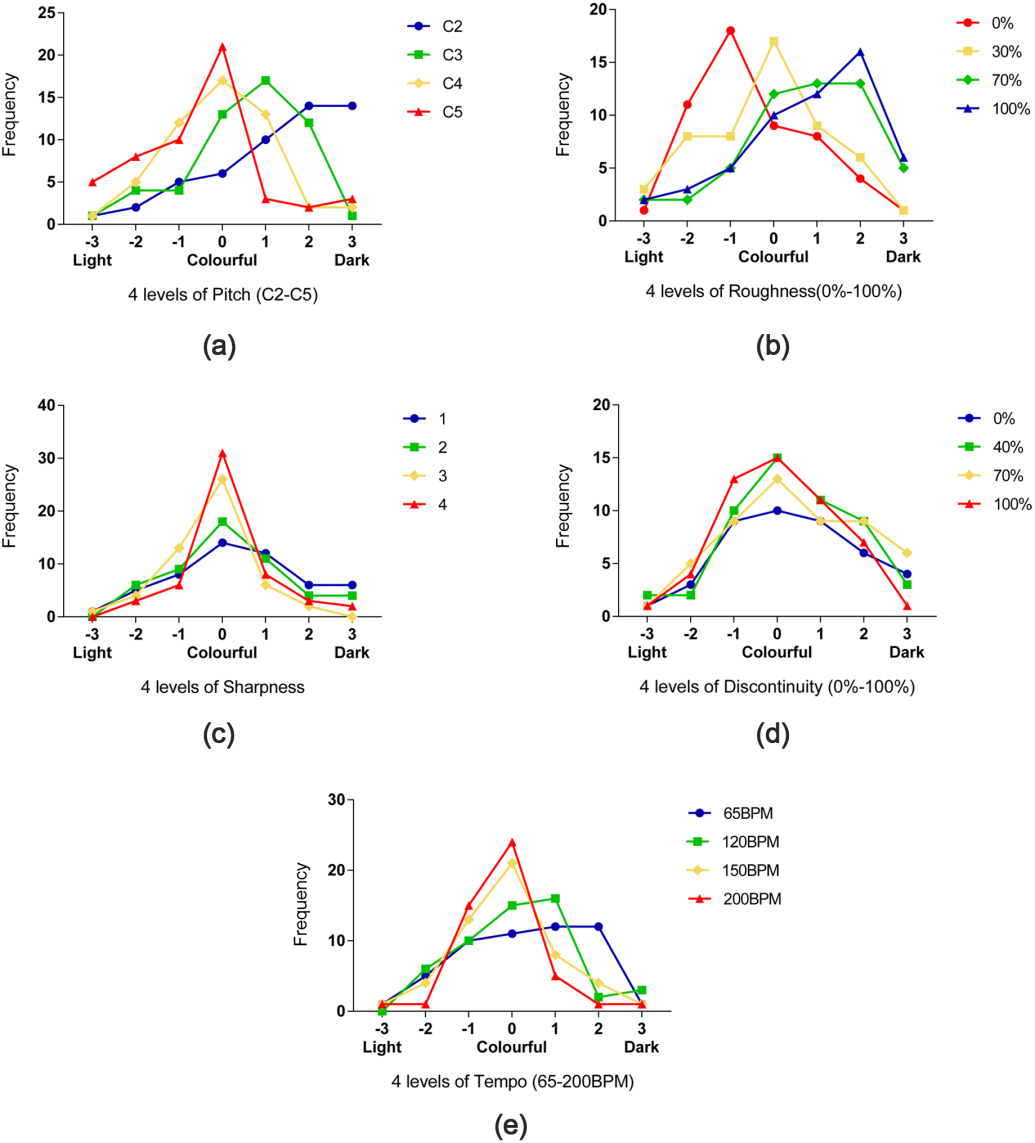
Results for sound-lightness mappings. Five plots (A–E) depicting the frequency with which the different colors (saturation and brightness) were selected for each of the five properties of sound (pitch, roughness, sharpness, tempo, discontinuity, in sequence). For each plot, different levels of lightness are shown along the *x*-axes, the corresponding values are shown along the *y*-axes. For example, in the “pitch” plot (A), four-line graphs represent the different data distribution in four levels of pitch, and higher pitch is associated with lighter colors, while lower pitch is more related to dark colors. Similarly, in the plot (E), fast tempo is also associated with lighter colors, slow tempo is related to dark colors. As shown in the plot (B), with the increase of roughness, participants are more tended to choose dark colors rather than light colors. In the plot (C), there’s a tendency that with the increase of sharpness, participants are inclined to choose more colorful. In the plot (D), color selections are found to be independent from different levels of discontinuity.

The results for sound-lightness mappings are shown in Fig. 5. For pitch (Fig. 5A), chi-square test of independence reveals that color selections are not independent from different levels [*χ*^2^(18) = 72.480, *p* < 0.001]. Results for post-hoc pairwise comparisons show significant differences exist between C2 and C3 [*χ*^2^(6) = 16.592, *p* = 0.007], C2 and C4 [*χ*^2^(6) = 27.820, *p* < 0.001], C2 and C5 [*χ*^2^(6) = 36.154, *p* < 0.001] and C3 and C5 [*χ*^2^(6) = 26.397, *p* < 0.001] (results for all possible combinations are listed in Table S2). Higher pitch was associated with lighter colors, while lower pitch was more related to dark colors.

For roughness (Fig. 5B), color selections are found to be associated with different roughness levels [*χ*^2^(18) = 43.745, *p* = 0.001]. There’re significant differences between 0 and 70% [*χ*^2^(6) = 22.692, *p* = 0.001] and between 0 and 100% [*χ*^2^(6) = 23.847, *p* = 0.001]. With the increase of roughness, participants were more tended to choose dark colors rather than light colors.

For sharpness (Fig. 5C), since the frequencies in three cells are zero, Fisher’s exact test is used to test the independence between variables. Results reveal that there’s a weak association between color selections and different sharpness levels (*p* = 0.045). There’s a tendency that with the increase of sharpness, participants are inclined to choose more colorful (higher saturation and brightness) colors. However, no significant difference is found between any possible pair in post-hoc test.

For discontinuity (Fig. 5D), chi-square test of independence reveals no significant difference between color selections and different levels [*χ*^2^(18) = 5.819, *p* = 0.448].

For tempo (Fig. 5E), results of Fisher’s exact of independence show that color selections are associated with different tempo levels [*χ*^2^(18) = 33.591, *p* = 0.004], where fast tempo appeared to be associated with the main colors, whereas slow tempo was related to dark colors. Post-hoc pairwise comparisons reveal significant differences between 65 BPM and 180 BPM (*p* < 0.001).

#### Reaction times

**Figure 6:**
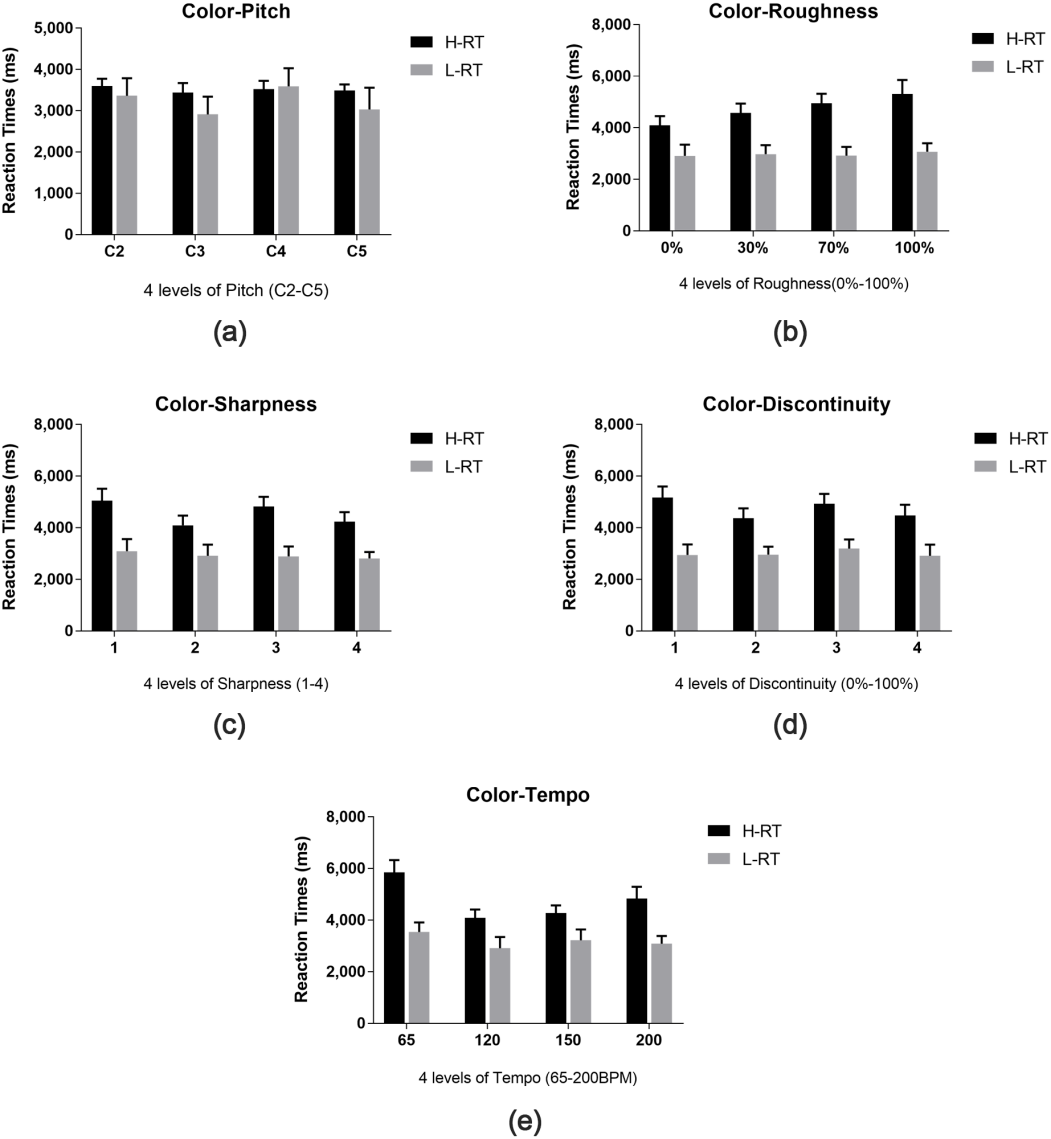
RTs for color-sound mappings. Five plots (A–E) depicting the time taken to assign hue and lightness for a given sound (four different levels of five properties). Five plots (A–E) describe the time taken to assign color for five different properties of sound respectively, which correspond to pitch, roughness, sharpness, tempo, discontinuity from A to E in sequence. For each plot, four levels of the corresponding property are shown along the *x*-axes. For legend, we denoted the response time to assign hue to sound as “H-RT” and the time taken to assign saturation or lightness to sound as “L-RT”, and the error bars indicate standard deviation of the reaction time.

The results for RTs are shown in Fig. 6. A 5 × 4 × 2 (sound attribute × level × color attribute) three-way repeated ANOVA is performed. Mauchly’s test of sphericity is performed and any data indicate a violation of sphericity were adjusted using Greenhouse-Geisser adjustment. Bonferroni’s *t*-test was also used in the post hoc test to identify where significance occurs.

The results reveal that there is a significant main effect for sound properties [*F*(4, 43) = 3.064, *p* = 0.018] and color properties [*F*(1, 46) = 60.161, *p* < 0.001]. There is also a significant sound attribute × color attribute interaction [*F*(2.91, 31.29) = 8.207, *p* < 0.001]. Irrespective of different levels and color attributes, RTs for hue-pitch mappings are significantly shorter than hue-tempo mappings (95% CI [−1,018.49-−70.68], *p* = 0.014). Irrespective of different sound attributes and levels, L-RTs are significantly shorter than H-RTs (95% CI [−1,777.12-−1,044.79], *p* < 0.001). Post-hoc analysis confirms that L-RTs are shorter than H-RTs in all sound attributes (roughness: 95% CI [−2,406.63-−1,314.03], *p* < 0.001; sharpness: 95% CI [−2,173.57-−1,176.55], *p* < 0.001; discontinuity: 95% CI [−2,089.26-−1,167.12], *p* < 0.001; tempo: 95% CI [−2,101.42-−1,096.68], *p* < 0.001), except for pitch (95% CI [−963.96–379.70], *p* = 0.386). Further analysis also revealed that H-RTs for pitch were significantly shorter than those for other sound attributes (roughness: 95% CI [−1,702.31-−628.80], *p* < 0.001; sharpness: 95% CI [−1,520.51-−512.51], *p* < 0.001; discontinuity: 95% CI [−1,739.96-−607.98], *p* < 0.001; tempo: 95% CI [−1,608.71-−787.38], *p* < 0.001), but no significant difference was found when comparing L-RTs for pitch to other sound attributes (roughness: 95% CI [−112.81–918.10], *p* = 0.123; sharpness: 95% CI [−233.41-−966.25], *p* = 0.225; discontinuity: 95% CI [−394.29–718.46], *p* = 0.560; tempo: 95% CI [−386.95–604.69], *p* = 0.661). No significant main effect was found for level [*F*(3, 44) = 2.150, *p* = 0.097]. There is also no interactive effect for sound attribute × level [*F*(5.78, 16.85) = 0.814, *p* = 0.556], level × color attribute [*F*(3, 44) = 1.513, *p* = 0.214] or sound attribute × level × color attribute [*F*(6.64, 19.37) = 1.564, *p* = 0.150].

### Discussion

In Experiment 1, we investigate the crossmodal correspondence of sound-hue and sound-lightness. In the study of correspondence between sound and color, participants match sound to color based on how they feel when they hear the sound. We extend previous research on the associations between sound properties and colors by investigating the psychoacoustic properties (roughness, sharpness and discontinuity) in addition to physical properties (pitch and tempo) of sound.

The results of Experiment 1 confirm pitch and tempo associate with color (hue, lightness, saturation). For example, high-pitch is associate with red, yellow and light-color, while low-pitch is associate with blue and dark-color. Furthermore, we find that roughness and sharpness are also related to color. Although chi-square test of reveals that roughness is independent to hue, post-hoc pairwise test finds significant difference between 0 and 100% levels, where purple and orange are associate with high roughness, while green and cyan are linked to low roughness. The insignificant overall association may be caused by the middle levels (30% and 70%), since the results of chi-square test of goodness of fit didn’t violate the randomness assumption at these two levels. We also find high roughness is significantly associated with low lightness in sound-lightness mappings. For sharpness, there’s a weak association between lightness and sharpness. Higher sharpness is linked to more colorful colors. To the best of our knowledge, this is the first study that finds the association between roughness and sharpness and color properties. Previous research has found that roughness and sharpness were associated with tastes (Knöferle & Spence, 2012; Knoeferle et al., 2015). Our findings can add to the literature that roughness and sharpness may also linked to visual aspects.

The result of RT is not significantly different between different sound properties or levels, but interestingly, participants make choice a little faster in sound-lightness mappings than in sound-hue mappings. There are two possible explanations for the differences: one is the potential practice effect, as participants always do sound-hue mappings followed by sound-lightness mappings. However, since participants have familiarized with all the 20 music pieces before experiment session and have familiarized with the same music piece as they listened to in sound-hue mappings and sound-lightness twice at the beginning of each trial, the practice effect is not likely to happen. A more reasonable explanation is that participants feel easier to match sound to lightness than to hue. Since the colors in sound-lightness are listed from light to dark, it may be more natural to link different levels of lightness to different levels of sound properties, while for sound-hue mappings, pure spectral colors with different hues are more like categorized options, which might make participants take more time to make a decision.

## Experiment 2

Experiment 2 is generally a revalidation experiment after Experiment 1. The aim of Experiment 2 is to reconfirm the correspondence between sound and color using a simple cognitive task, and assess whether the result of the cognitive task is consistent with the result in Experiment 1. We hypothesize that the correspondence between sound and color would impact on response latencies in a simple cognitive task.

### Materials and methods

#### Participants

Twenty-two participants (11 males and 11 females, Mage = 22.95, SD age = 1.889, range 20–30 years) are chosen from Experiment 1 to take part in the Experiment 2. In Experiment 2, we design a speeded target discrimination task which includes a memory task. Considering that memory is related to age, in the selection of participants, only young participants whose ages range from 20 to 30 are included in Experiment 2. Participants are randomly divided into two equal groups—Group A and Group B. Participants from each group are required to finish three sets of cognitive tasks: Group A (Congruent sound-hue, Congruent sound-lightness, Control A); Group B (Incongruent sound-hue, Incongruent sound-lightness, Control B). The type of consistency to be discriminate (congruent pairing and incongruent pairing) is tested in two different groups in order to avoid any conflict in participants’ memory. The participants also give their written informed consent before the start of the experiment. Two participants are excluded from further analysis due to technical reasons. Thus, only 20 participants are included in the data analyses.

#### Apparatus and materials

The apparatus and materials used in this experiment are identical to those used in Experiment 1.

#### Stimuli

The sound stimuli are selected from those used in Experiment 1 on the basis that they have been found to be associated with certain colors. Since pitch has been found to have the strongest association with color among the five sound attributes, we specially chose pitch to focus on in Experiment 2. According to the results of Experiment 1, red and light colors are associated with high pitches, while blue and dark colors seem to be linked to low pitches. Therefore, two waveform sounds, high pitch (523 Hz) versus low pitch (130 Hz), are used in this experiment. Two pairs of typical colors with different hue or lightness include red (FF0000) versus blue (0000FF) and light grey (F3F3F3) versus dark grey (262626) are used to make congruent or incongruent pairings with the sound stimuli for sound-hue pairings and sound-lightness pairings, respectively. Green (00FF00) and Cyan (00FFFF), both of which show no association with pitch in Experiment 1, are also used as the control treatment (Control A and Control B).

#### Procedure

**Figure 7:**
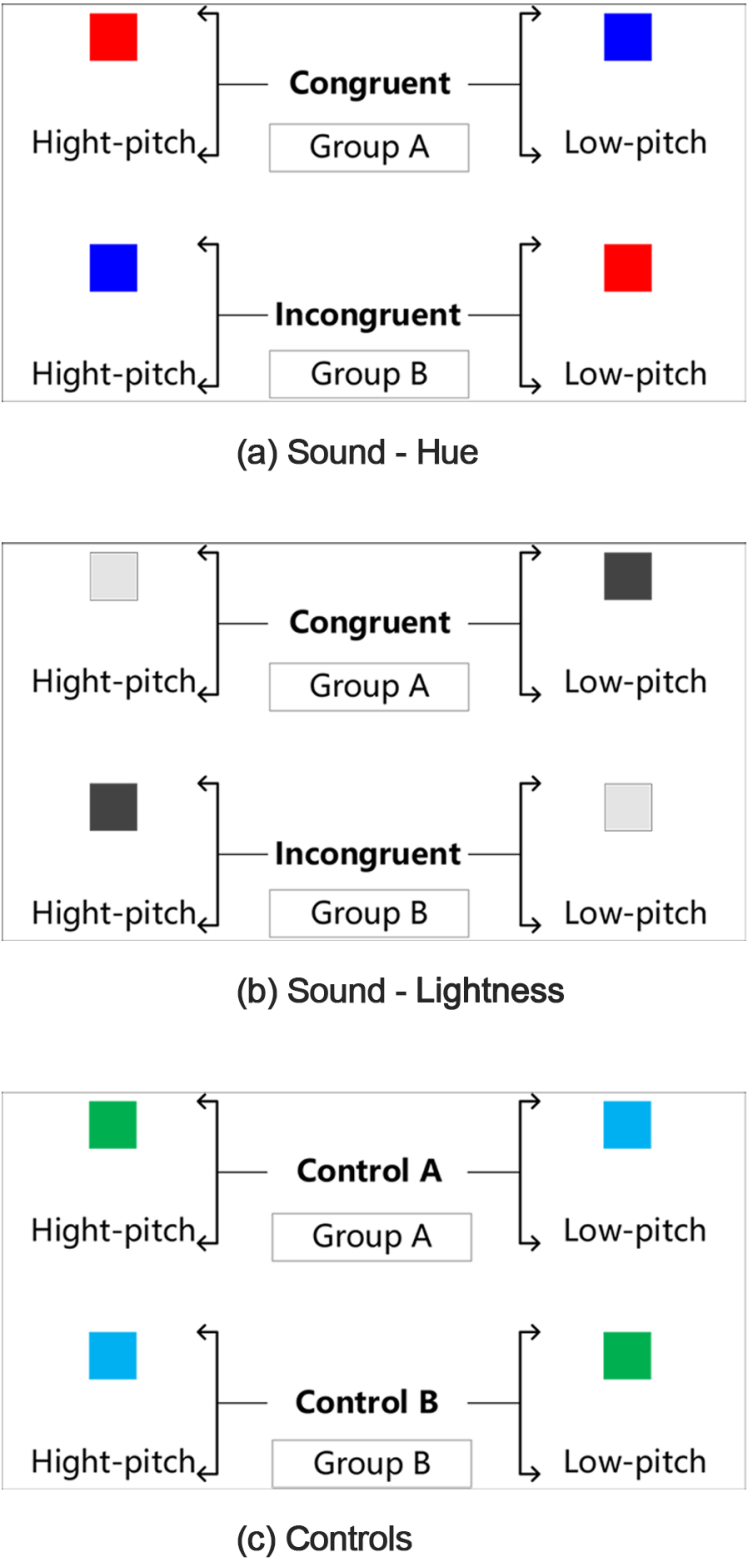
The experimental protocol of the speed discrimination task. Three plots provide a graphical illustration of the pairs in (A) sound-hue, (B) sound-lightness, (C) Control. For each plot, two groups (Group A and Group B) are required to memorize the different predefined sound-color pairings. For example, in plot (A), Group A regarded “high pitch with red—congruent pairing” as “correct” pairing, whereas group B regarded “high pitch with blue—incongruent pairing” as “correct” pairing. Participants were instructed to discriminate the target color as rapidly and accurately as possible.

Experiment 2 is conducted about one month after the completion of Experiment 1. Stimuli and operation are presented using E-prime software. Participants from both groups are required to memorize the predefined sound-color pairings, i.e., which two are “Correct” pairings, which two are not. All predefined pairings are shown in Fig. 7. The sound-color pairings in two groups are predefined in the opposite way. In Group A, we define the **congruent** sound-color pairings which have higher relevance as the “Correct” pairings. For example, high pitch with red or light grey, low pitch with blue or dark grey. Instead, in Group B, **incongruent** sound-color pairings are defined as the “Correct” pairings. For example, high pitch sound with blue, low pitch with red or light grey. For the **control** treatment, in Control A, high pitch with green and low pitch with cyan are defined as “Correct” pairings. In Control B, “Correct” pairings are defined in the opposite way. All predefined pairings are shown in Fig. 7.

**Figure 8:**
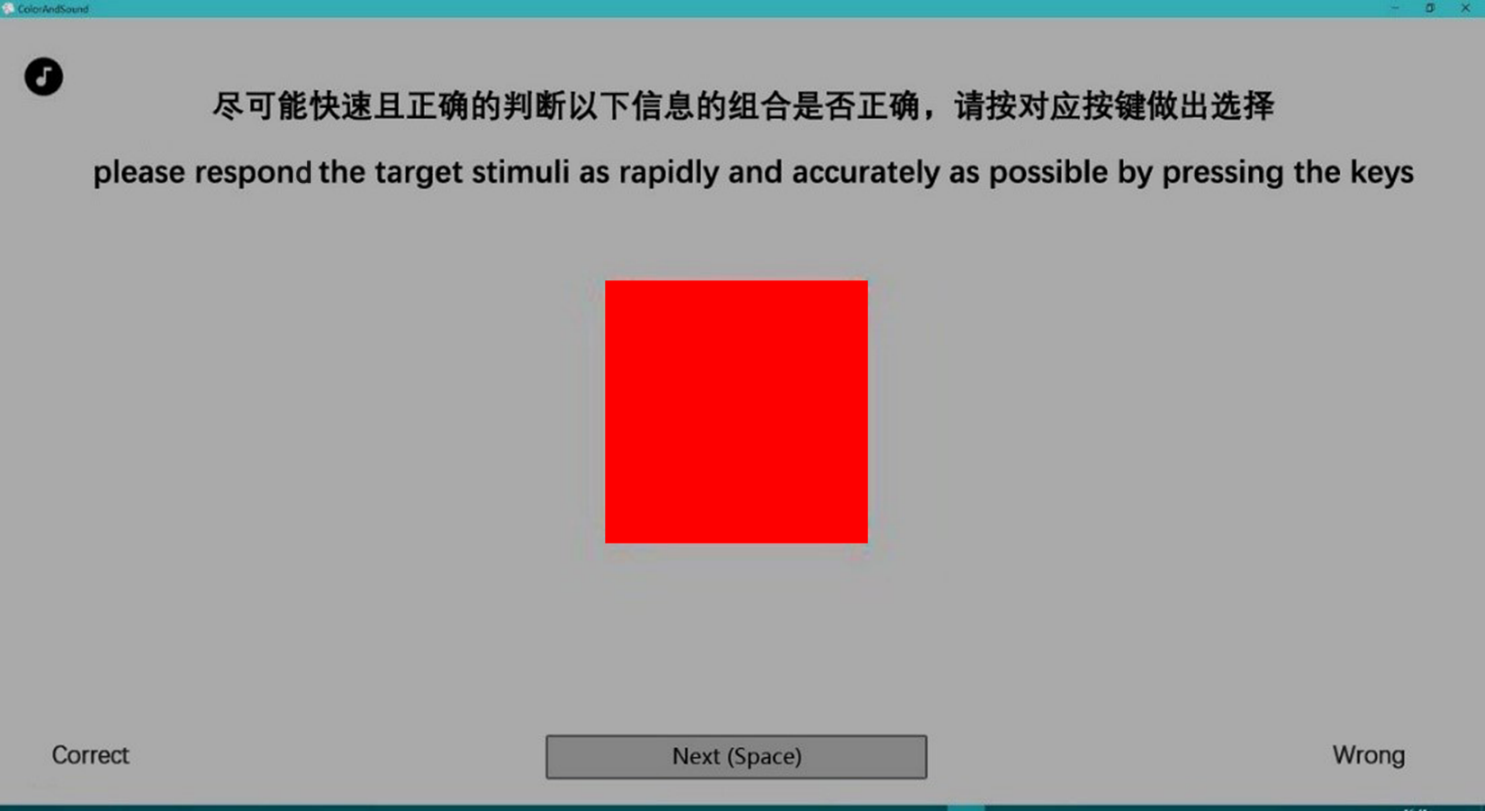
Screenshot illustrating the task used in Experiment 2. Participants were instructed to discriminate the target color as rapidly and accurately as possible by pressing the keys (the “q” and “p” keys). Participants made responses with the keyboard. For example, when a red color square is shown with high-pitch tone presented over the headphone, participants in Group A should judge it was the “correct” pairing as rapidly as possible by pressing the “q” key, while Group B should judge it was the “incorrect” pairing and press the “p” key.

Participants are asked to be seated in front of a computer and wear a headphone. Before the experimental session begins, participants are asked to listen to the sound stimuli for familiarization. Each participant also has 15 min to memorize the predefined sound-color pairings. Participants are also given instruction and practice mode prior to the trials. They were told that they would hear some sounds presented over the headphones. After completing their memorizations, participants are required to play a simple puzzle game—Magic Puzzles, the purpose of which is to distract participants’ attention instead of performing the speeded target discrimination task directly after memory. Then the experimental session begins. The given sound is presented for 2 s prior to the presentation of the target color. Participants are instructed to discriminate the target color as rapidly and accurately as possible by pressing the keys (“q” and “p”). They are instructed to choose “Yes” by pressing the “q” key when it is the “correct” pairing, and to choose “No” by pressing the “p” key when it is the “incorrect” pairing. For example, when it is a high-pitch tone followed by red color, Group A should judge it is the “correct” pairing as rapidly as possible by pressing the “q” key, while Group B should judge it is the “incorrect” pairing and press the “p” key. Target stimuli persist until participants make a decision by pressing the key (“q” and “p”) and the next trail starts after 2 s pause. Each of the possible pairings (e.g., red/high-pitch, blue/low-pitch, red/low-pitch, blue/high-pitch) would repeat for five times. Stimuli present randomly in the experiment. Each group have to accomplish 60 trails which are consisted of three treatments—sound-hue pairings, sound-lightness pairings and control treatment. Each trail begins with the presentation of a fixation point in the center of the screen for an interval of 500 ms. Screenshot for one of the trails is shown in Fig. 8.

### Results

#### Reaction time

**Figure 9:**
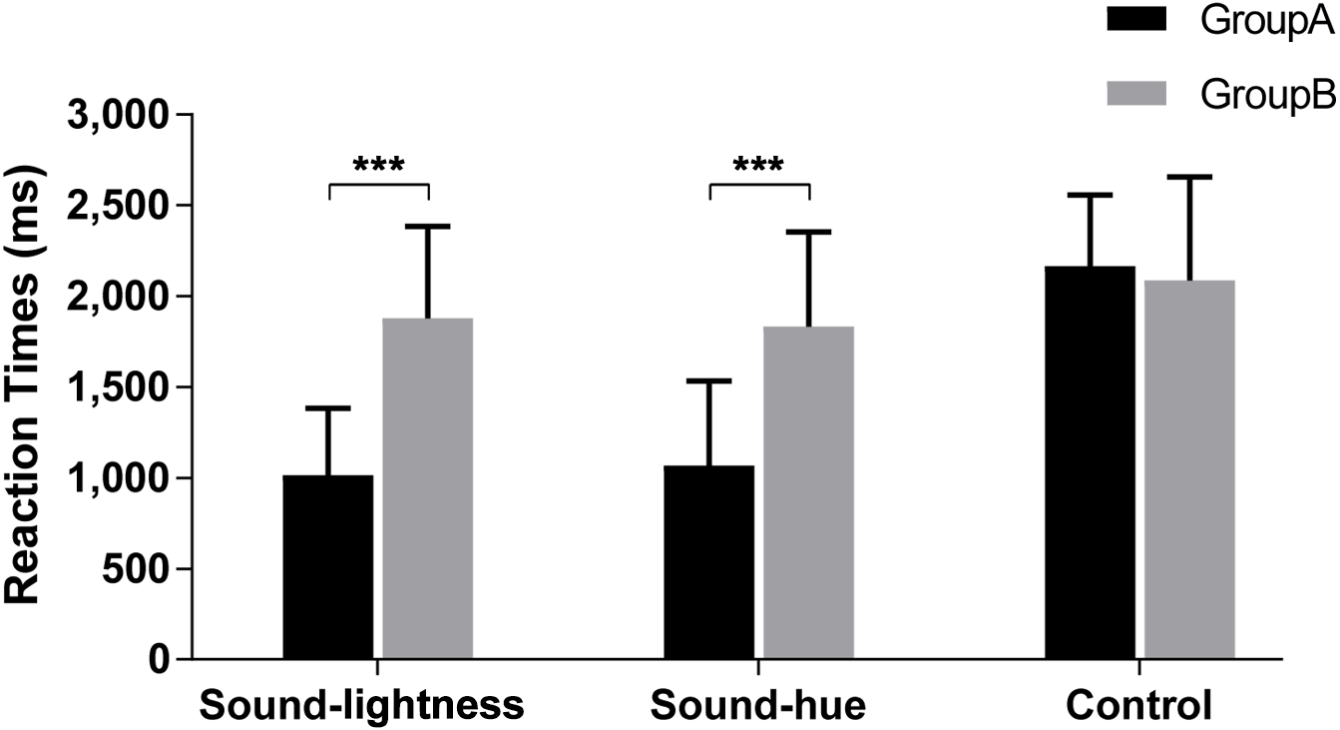
RTs for speed discrimination task. Group A: Congruent; Group B: Incongruent. *** indicate statistical significance of *p* < 0.001, and the error bars indicate standard deviation of the reaction time.

RTs were measured from the onset of the given stimulus. The conclusive evidence is depicted in Fig. 9. Two-way (group × treatment) repeated measures ANOVA reveals that both group [*F*(1, 18) = 26.177, *p* < 0.001] and treatment (sound-lightness, sound-hue and control) [*F*(1.339, 24.102) = 241.052, *p* < 0.001] have significant effects on the RTs for speed discrimination. Significant group × treatment interaction is also found [*F*(1.339, 24.102) = 105.801, *p* < 0.001]. Pairwise comparisons reveal that RTs are shorter in Group A than Group B for both sound-lightness (95% CI [−1,016.82-−510.60], *p* < 0.001) and sound-hue (95% CI [−1,075.40-−653.48], *p* < 0.001) treatments, whereas no significance is found for control treatment (95% CI [−142.00–299.54], *p* = 0.463). These results indicate that, in non-synesthetes, the effect of sound-color congruency can influence their performance in speeded discrimination, which is analogous to the weak-Stroop effect.

Irrespective of groups, there’s no significant difference between sound-lightness and sound-hue (95% CI [−50.36–59.54], *p* = 1.000). However, RTs in both sound-lightness (95% CI [−791.40-−558.08], *p* < 0.001) and sound-hue (95% CI [−778.64-−580.03], *p* < 0.001) are found to be shorter than in control, even for Group B (sound-lightness and control: 95% CI [−384.84-−122.16], *p* = 0.001; sound-hue and control: 95% CI [−319.52-−95.93], *p* < 0.001).

#### Errors

**Figure 10:**
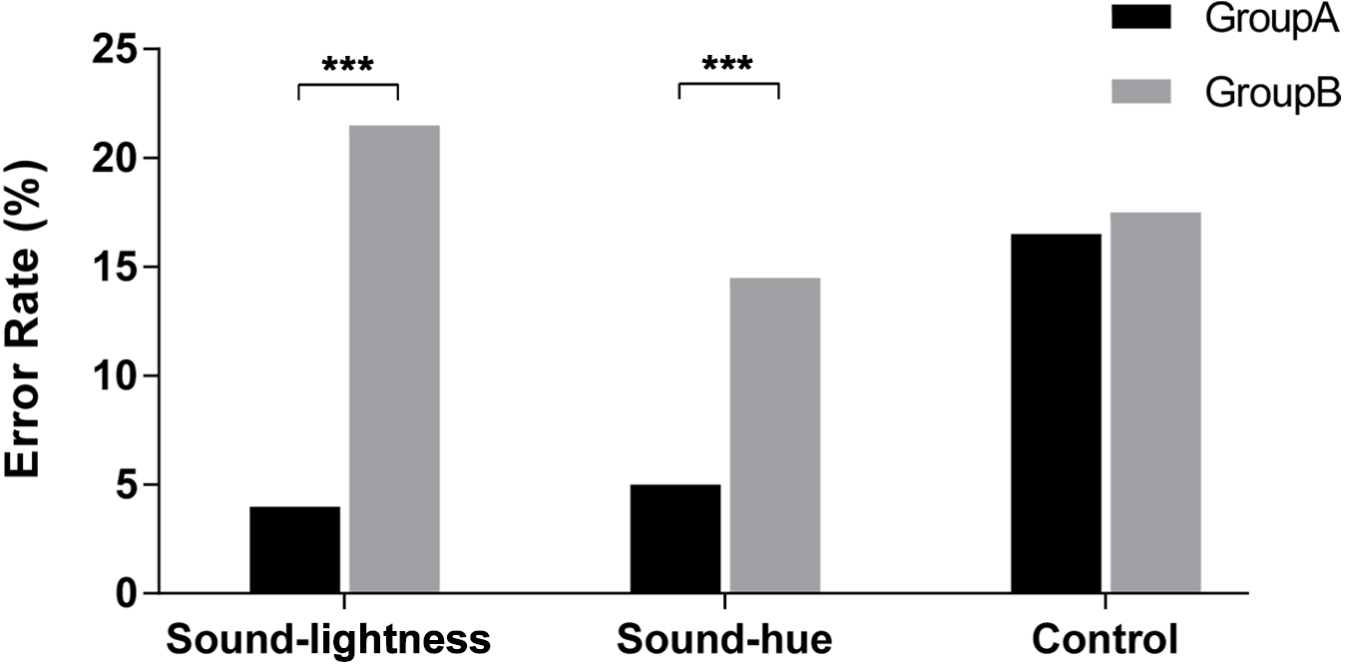
Error rates for speed discrimination task. Group A: Congruent; Group B: Incongruent. *** indicate statistical significance of *p* < 0.001.

Results for error rates are shown in Fig. 10. Error data are relatively scant as many participants make none or virtually none. Hence, changes in error rates are generally not reliable within individual conditions. Therefore, we accumulate the error frequencies of all participants in the same treatments and groups, and compare the differences between different treatments. Chi-square test is used to compare the difference of error rate and correct rate and to identify whether there is difference between Group A (Congruent) and B (Incongruent) in each treatment. Results reveal the existence of significant interaction of congruency in both sound-lightness and sound-hue treatments. Error rates are significantly higher in Group B than Group A (for sound-lightness: *χ*^2^(1) = 27.530, *p* < 0.001; for sound-hue, *χ*^2^(1) = 10.462, *p* = 0.001). No significance is found between Group A and Group B (*χ*^2^(1) = 0.018, *p* = 0.893).

Comparing the error rates in Group, it seems that there was a tendency that error rates in sound-lightness are much higher than in sound-hue. However, results reveal that the difference between these two treatments is not significant (*χ*^2^(1) = 3.320, *p* = 0.068). For congruent condition, no significance is found between sound-lightness and sound-hue (*χ*^2^ = 0.233  *p* = 0.630).

### Discussion

In Experiment 2, we focus on studying the impact of sound-color correspondence on a cognitive task, which involves a speeded target discrimination to assess whether participants could invoke naturally crossmodal correspondence. Pitch of the sound properties and red and blue colors are specially chosen for they were found to have the strongest sound-hue association in Experiment 1. Light and dark grey are used for sound-lightness pairings.

The result of Experiment 2 reveals that there are significant differences on RTs and error rates between congruent and incongruent, both RTs and errors of congruent group (Group A) is much lower than that of incongruent group (Group B). The strong connection between pitch and hue and between pitch and brightness have counterparts in speeded discrimination, with responses to high (or low) pitch being faster and more accurate when accompanied by red/light-grey (or blue/dark-grey). This result further supports the autonomic association between sound and color in non-synesthetic individuals. It also demonstrates that crossmodal correspondence does have an influence on cognitive task. In addition, control treatment is also set and we find that the RTs in controls (both Group A and Group B) are higher than those in congruent group of sound-hue treatment and sound-lightness treatment. This phenomenon may have some referential value for the design of multimodal man-machine interface. An interesting finding is that in incongruent groups, participants react faster in control treatment than in sound-hue or sound lightness treatment. We speculate this is caused by an effect of reverse memory, at least a certain inverse relationship is established in incongruent pairs, which make the participants memorize the incongruent pairs more easily.

## General Discussion

In this study, Experiment 1 investigates the crossmodal correspondence between sound and hue and between sound and lightness. Instead of using simple stimulus dimensions, such as pitch and color-words, or more complex stimuli, such as pictures and music, in the present study, we manipulate twenty sounds with five auditory properties, each varies in four levels, to assess whether these attributes have an influence on the association. Forty-nine colors with different hues, saturation and brightness are also selected to match the sounds. Experiment 2 are concerned with the automaticity and effect of crossmodal correspondence, comparing the difference between sound-hue and sound-lightness in the speed discrimination task,

The results of experiment 1 demonstrate the “one-to one” association between each sound property and each color property in non-synesthetic participants. Results confirm the existence of clear correspondence relations between sound and hue and sound and lightness. Certain color properties are found to be easier for people to associate with certain sound, which indicates that these properties may be better suited to conveying certain sound attributes. For sound and hue, high pitch and fast tempo are generally associated with red and yellow colors, whereas low pitch and slow tempo are related to blue and orange. Our finding is consisted with previous research (Marks, 1974; Hagtvedt & Brasel, 2016). In addition, the results demonstrate that other sound attributes besides pitch and timbre, such as roughness and sharpness, also have influence on the association between sound and color. Roughness is generally associate with green, cyan, purple, orange, but not with red and yellow; Sharpness has significant association with red and cyan. For sound and lightness, higher pitch and fast tempo seem to be more related to pure spectral colors or lighter colors, while low-pitch and slow tempo seem to be most strongly associated with dark color, which is in line with previous findings. We find that roughness and sharpness are also associated with lightness. With the increase of roughness, the associated color gradually changes from dark towards light. With the increase of sharpness, pure spectral colors are more frequently chosen by participants.

For RTs in the result of Experiment 1, we find that there is no difference of RTs among different types or levels of some sound properties, such as pitch and tempo, low-pitch and high-pitch. However, there is a little difference on RTs between sound-hues and sound-lightness. RTs is a little larger when participants respond to the hues for the given sounds than to lightness. That is, participants feel easier when they are matching sound with lightness rather than hues, which is an interesting finding that came to our attention. Thus, Experiment 2 is designed to assess the difference between sound-hue and sound-lightness using a speed discrimination task.

A forced-choice matching task which involves a speeded target discrimination to assess whether participants could invoke naturally crossmodal correspondence, which is suited for comparing the difference between sound-hue and sound-lightness. The results of Experiment 2 further support the association between sound and color in non-synesthetic participants. Participants in congruent group respond much quicker than in incongruent group. It also demonstrates that crossmodal correspondence has obvious interference on cognitive memory tasks under incongruent conditions. However, RT data show that there is no significant difference between sound-hue and sound-lightness. The inconsistency between the results of Experiment 1 and 2 probably lies on the difference of the experimental protocols. As we have discussed in Experiment 1, we speculated that L-RT was a little shorter than H-RT because participants felt easier to link different lightness levels to different levels of sound properties than the “categorized” hues. In Experiment 2, the experiment protocol of sound-hue pairings and sound-lightness pairings are same. The insignificant result may support our hypothesis to some extent.

In the present study, there are several potential limitations that should be mentioned. First, the piano chord we use in the Experiment 1 may more or less influence the results. Every musical sound has its own emotional influence, which is even inevitable although we manipulate the sounds with five properties based on the same chord. Second, the different complexity of the experimental designs results in inconsistency between our two experiments, which needs to be further investigated. Third, although we have demonstrated that pitch, tempo, roughness and sharpness are associated with hue and lightness, it is generally unclear which properties have the biggest influence on the association of sound and color. We will devote ourselves to answering this question in future.

## Conclusions

In conclusion, the present study is designed to investigate whether various sound attributes, such as pitch, tempo, roughness, sharpness, have varying degrees of influence on the crossmodal correspondence with each attribute of color, and to assess the impact of crossmodal correspondence on human information processing using the speeded classification task. Our findings replicate and extend previous research. Results of experiment 1 show that pitch, tempo, roughness and sharpness are associated with hue and brightness. Participants respond to the lightness for the given sounds a little more quickly than to hue. The results of Experiment 2 confirm that cognitive responses to color or sound are influenced by the sound-color correspondence. People respond to high (or low) pitch much faster and more accurate when accompanied by red/light-grey (or blue/dark-grey). There is no difference between sound-hue and sound-lightness, which is probably owing to the different experimental design to Experiment 1. Our findings highlight the existence of crossmodal correspondence between sounds and colors in psychology and the effects on cognition task.

##  Supplemental Information

10.7717/peerj.4443/supp-1 Datset S1 Dataset of Experiment 1

10.7717/peerj.4443/supp-2 Dataset S2 Dataset of Experiment 2

10.7717/peerj.4443/supp-3 Table S1 Results for post-hoc test of sound-hue mappings Results for post-hoc pairwise comparisons of all possible combinations for pitch, roughness and tempo with hue. Alpha level is set at *α* < 0.0083.

10.7717/peerj.4443/supp-4 Table S2 Results for post-hoc test of sound-lightness mappings Results for post-hoc pairwise comparisons of all possible combinations for sound-lightness mappings. Alpha level is set at *α* < 0.0083.

## Additional Information and Declarations

### Data Availability

The following information was supplied regarding data availability: The raw data is provided in the Supplemental Files.
