# Supplementary material for: An extended research of crossmodal correspondence between color and sound in psychology and cognitive ergonomics
Source: PeerJ. 2018 Mar 1;6:e4443. doi: 10.7717/peerj.4443 (PMC5835347; doi:10.7717/peerj.4443)
Supplement: Table S1 — Results for post-hoc pairwise comparisons of all possible combinations for pitch, roughness and tempo with hue. Alpha level is set at α < 0.0083. [file peerj-06-4443-s003.docx]

|  | **χ^2^(6)** | | **p** |
| --- | --- | --- | --- |
| **Pitch** | | | |
| C2 vs C3  **C2 vs C4**  **C2 vs C5**  C3 vs C4  C3 vs C5  C4 vs C5 | | 11.477  **23.053**  **34.906**  3.379  9.828  3.494 | 0.075  **0.001**  **< 0.001**  0.760  0.132  0.745 |
| **Roughness** | | | |
| 0 vs 30%  0 vs 70%  **0 vs 100%**  30 vs 70%  30 vs 100%  70 vs 100% | | 2.473  8.103  **19.500**  2.392  9.188  4.684 | 0.872  0.231  **0.003**  0.880  0.163  0.585 |
| **Tempo** | | | |
| 65 vs 120 BPM  65 vs 150 BPM  **65 vs 180 BPM**  120 vs 150 BPM  120 vs 180 BPM  150 vs 180 BPM | | 4.635  10.129  **23.557**  1.665  8.937  5.162 | 0.591  0.119  **0.001**  0.948  0.177  0.523 |
