# Supplementary material for: An extended research of crossmodal correspondence between color and sound in psychology and cognitive ergonomics
Source: PeerJ. 2018 Mar 1;6:e4443. doi: 10.7717/peerj.4443 (PMC5835347; doi:10.7717/peerj.4443)
Supplement: Table S2 — Results for post-hoc pairwise comparisons of all possible combinations for sound-lightness mappings. Alpha level is set at α < 0.0083. [file peerj-06-4443-s004.docx]

|  | **χ^2^(6)** | | **p** |
| --- | --- | --- | --- |
| **Pitch** | | | |
| **C2 vs C3**  **C2 vs C4**  **C2 vs C5**  C3 vs C4  **C3 vs C5**  C4 vs C5 | | **16.592**  **27.820**  **36.154**  12.654  **26.397**  10.412 | **0.007**  **< 0.001**  **< 0.001**  0.049  **< 0.001**  0.102 |
| **Roughness** | | | |
| 0 vs 30%  **0 vs 70%**  **0 vs 100%**  30% vs 70%  30% vs 100%  70% vs 100% | | 8.240  **22.962**  **23.847**  11.327  13.492  0.786 | 0.221  **0.001**  **0.001**  0.074  0.036  0.992 |
| **Sharpness**  Level 1 vs Level 2  Level 1 vs Level 3  Level 1 vs Level 4  Level 2 vs Level 3  Level 2 vs Level 4  Level 3 vs Level 4 | |  | |
|  |  | -*  -  -  -  -  - | 0.918  0.012  0.019  0.127  0.162  0.431 |
| **Tempo** | | | |
| 65 vs 120 BPM  65 vs 150 BPM  **65 vs 180 BPM**  120 vs 150 BPM  120 vs 180 BPM  150 vs 180 BPM | | -  -  **-**  -  -  - | 0.080  0.145  **< 0.001**  0.304  0.011  0.624 |

* chi-square value is not applicable for Fisher’s exact test.
